# Targeting dementia prevention with semaglutide: The case for *APOE4* homozygotes

**DOI:** 10.1002/alz.70422

**Published:** 2025-06-26

**Authors:** Timothy Daly, Bruno P. Imbimbo

**Affiliations:** ^1^ UMR 1219 Bordeaux Population Health Université de Bordeaux & INSERM Bordeaux France; ^2^ UMR 5164 ImmunoConcept University of Bordeaux & CNRS Bordeaux France; ^3^ Bioethics Program FLACSO Argentina Buenos Aires Argentina; ^4^ Research & Development Chiesi Farmaceutici Parma Italy

1

In *Alzheimer's & Dementia*, Wang et al. used a target trial emulation approach based on U.S. electronic health records and reported a significant reduction in the first‐time diagnosis of Alzheimer's disease (AD) among patients with type 2 diabetes mellitus (T2DM) treated with semaglutide.^1^ While their findings warrant cautious interpretation due to the potential for unmeasured confounding,^2^ they contribute to a growing body of evidence—including other target trial emulations^3^ and systematic reviews and meta‐analyses of randomized controlled trials (RCTs)^4^—supporting the neuroprotective effects of semaglutide.

Importantly, differences in the degree of neuroprotection appear to exist both within the class of glucagon‐like peptide‐1 receptor agonists (GLP‐1 RAs), to which semaglutide belongs,^3^ and between GLP‐1 RAs and other cardioprotective glucose‐lowering therapies.^4^ This suggests that semaglutide's neuroprotective effects are unlikely to be mediated solely by glycemic control, supporting its potential as a neuroprotective agent even in populations without T2DM. Experts commenting on recent findings involving antidiabetic drugs in dementia^5^ have emphasized the need for RCTs to mitigate the risks of false positives (i.e., incorrectly concluding a protective effect) and false negatives (i.e., failing to detect a true benefit) inherent in observational studies. One such trial is the ongoing EVOKE and EVOKE+ study, which evaluates semaglutide versus placebo over 3 years in individuals with early symptomatic AD.^6^

However, the history of AD research has highlighted the importance of presymptomatic interventions and the slow progression to dementia along the AD continuum.^7^ To generate high‐quality data on the neuroprotective effects of semaglutide in dementia prevention among individuals without T2DM, studies should target populations with a non‐diabetic genetic risk profile in which conversion to dementia within a normal lifespan is certain or highly probable.

The small population with autosomal dominant AD could serve as one such model,^8^ although recruitment would be limited by the rarity of the condition. In contrast, apolipoprotein E4 (*APOE4)* homozygotes—who comprise approximately 2% of the general population—represent a group that develops dementia, on average, 7–10 years earlier than non‐carriers.^9^ Furthermore, this population faces an elevated risk of developing serious amyloid‐related imaging abnormalities (ARIA) with United States Food and Drug Administration (FDA)‐approved lecanemab, rendering them ineligible for treatment in healthcare systems such as the European Union (EU), where lecanemab is contraindicated in *APOE4* homozygotes. We therefore advocate for RCTs testing the neuroprotective effects of semaglutide in cognitively unimpaired older adults who are *APOE4* homozygotes and exhibit biomarkers indicative of increased neurodegenerative burden and accelerated cognitive decline.^10^

In conclusion, trials of semaglutide in *APOE4* homozygotes would enable a feasible and high‐quality assessment of its therapeutic potential in a high‐risk population with limited treatment options, while also providing a clearer indication of its neuroprotective effects in the broader population.

## CONFLICT OF INTEREST STATEMENT

Dr. Timothy Daly has no conflicts of interest to declare. Dr. Bruno P. Imbimbo is an employee at Chiesi Farmaceutici. He is listed as an inventor in a number of Chiesi Farmaceutici's patents of anti‐Alzheimer drugs. Author disclosures are available in the Supporting Information.

## Supporting information



Supporting Information
